# Phase I study of imatinib, cisplatin and 5-fluoruracil or capecitabine in advanced esophageal and gastric adenocarcinoma

**DOI:** 10.1186/1471-2407-12-587

**Published:** 2012-12-10

**Authors:** Martina Mayr, Karen Becker, Nadine Schulte, Sebastian Belle, Ralf Hofheinz, Annekatrin Krause, Roland M Schmid, Christoph Röcken, Matthias P Ebert

**Affiliations:** 1 Department of Internal Medicine II, Klinikum Rechts der Isar, Technical University of Munich, Munich, Germany; 2 Institute for Pathology, Klinikum Rechts der Isar, Technical University of Munich, Munich, Germany; 3 Department of Medicine II, University Hospital Mannheim, Ruprecht-Karls-Universität Heidelberg, Heidelberg, Germany; 4 Department of Medicine III, University Hospital Mannheim, Ruprecht-Karls-Universität Heidelberg, Heidelberg, Germany; 5 Novartis Pharma GmbH, Nürnberg, Germany; 6 Institute of Pathology, Christian-Albrechts Universität, Kiel, Germany

**Keywords:** Imatinib, PDGF, Gastric cancer, Chemotherapy

## Abstract

**Background:**

Despite all benefit provided by established therapies prognosis of gastric cancer remains poor. Targeted inhibition of platelet derived growth factor receptor (PDGFR) by imatinib may influence tumor growth and amplify chemotherapeutic effects.

**Methods:**

This phase I study evaluated dose limiting toxicity (DLT) of imatinib combinated with chemotherapy according to a 3-patient cohort dose-escalating design. Thirty-five patients received cisplatin (60 mg/m^2^ d1 q 3w)/ capecitabine (1250 mg/m^2^ bid d1-14 q 21) or cisplatin (50 mg/m^2^ d1 q 2w)/ 5-fluoruracil (2 g/m^2^ d1, q 1w). Imatinib was started d - 4 with dose escalation from 300 to 700 mg QD in 100 mg steps.

**Results:**

At imatinib dose level 1 (300mg) one DLT was observed, three more patients were enrolled without further DLT. At dose level 5 (700 mg) two gastric perforations occurred, so 600 mg imatinib emerged as the maximum tolerated dose. Major grade 3/4 toxicities were nausea (6%), anemia (6%) and fatigue (3%). Response evaluation revealed partial response in 27% and stable disease in 43% of the assessable patients.

**Conclusions:**

Combination of imatinib and chemotherapy is well tolerated. Response rates were not superior to those of standard therapy. Further investigations of a larger group of patients are required to confirm the amplification of chemotherapy effects by imatinib.

**Trial registration:**

European Clinical Trials Database: Eudra-CT2006-005792-17 and Clinical Trials Database: NCT00601510

## Background

Evidence for benefit of chemotherapy in patients with advanced gastroesophageal and gastric cancer is increasing. Several studies demonstrated improved overall survival and quality of life for chemotherapy compared to best supportive care [1-3]. Also, a variety of clinical trials showed superiority of combination therapy over single-agent therapy [4]. But resection is still the only chance for cure and most patients present with advanced disease initially. Despite all positive effects of pre- and perioperative chemotherapy, relapse rates of gastric cancer are high. Integration of targeted therapies into novel treatment strategies may provide an additional benefit, but actually experience in this field is limited to just a few trials [5].

Receptor tyrosine kinases (RTK) represent an interesting molecular target due to their pivotal role in signal transduction and malignant transformation. They have an essential function in the regulation of cell growth, development, metastasis and apoptosis [6-8]. The platelet-derived growth factor receptors (PDGFR) as a transmembrane RTK subgroup are expressed in various human tumors, including ovarian, gastric and colorectal cancer [9-12]. The platelet-derived growth factors (PDGF) proteins constitute several isoforms: PDGF-AA, PDGF-BB, PDGF-AB, PDGF-CC and PDGF-DD [13]. The PDGF α-receptor binds all variants except PDGF-DD, whereas the β-receptor binds only PDGF-BB. PDGFs are potent chemotactic and mitogenic growth factors for fibroblasts, endothelial cells and smooth muscle cells. They stimulate the growth of stromal tissue in malignant tumors by activating so called carcinoma-associated fibroblasts (CAFs) [14]. Besides they have been found to promote angiogenesis [15], to recruit pericytes [16] and to affect interstitial fluid pressure (IFP) being responsible for the transvascular transport of chemotherapeutics [17]. Therefore inhibition of the PDGF RTK- signaling promises interesting effects on tumor growth.

Imatinib (Glivec ®) is a highly selective inhibitor of the RTK family comprising Abl, the Bcr-Abl fusion protein found in most cases of chronic myeloid leukemia (CML), PDGFR- α and -β, and the product of the c-kit proto-oncogene (KIT). In patients with CML and in patients with c-kit-positive gastrointestinal stroma tumors imatinib therapy is a well tolerated and effective treatment strategy. Several trials demonstrated the pivotal role of PDGF-B and PDGFR-β expression for human gastric carcinoma as well in mouse models [8] as in gastric carcinoma cell lines and surgical specimens [18]. The authors presume promising effects by the blockade of PDGFR signaling pathways. According to this, our own preclinical data demonstrate gastric cancer cells expressing PDGFR as well as c-kit and their growth being evidently inhibited by imatinib. Further, previous studies showed imatinib to amplify the effects of cisplatin [7] and 5-fluoruracil and leucovorin [19] in certain cancer cells. Therefore blocking the PDGF mediated signal transduction pathway may enhance chemotherapeutic effects in the treatment of gastric cancer. In mouse models especially combination with high-dose imatinib revealed significant potency [8].

This phase I study was performed to evaluate safety and dose limiting toxicity (DLT) of imatinib in combination with chemotherapy in patients with advanced esophageal and gastric adenocarcinoma. Besides docetaxel-based regimens and trastuzumab containing therapies for HER2-positive cancer, 5-fluoruracil (FU)-based combinations with cisplatin or oxaliplatin are regarded the chemotherapeutic standard. The PLF- regimen with 5-FU (F), leucovorin (L) and cisplatin (P) is one of the most common regimes [3,20]. However, 5-FU is replaced more often by its prodrug Capecitabine, since recent studies proved non-inferiority of capecitabine compared to infusional FU and similar toxicities except diarrhea and hand-foot syndrome [21-23]. Therefore both fluoropyrimidines were combined with cisplatin and imatinib in order to assess the toxicity of this therapy in gastric and esophageal adenocarcinoma patients.

## Methods

### Preclinical analysis

Preliminary investigations of the PDGFR– and c-kit (CD 117) - expression in gastric cancer cells were performed on tissue specimens of 57 patients with gastric cancer (specimens were retrieved from the archive of the department of pathology, Kiel). The average age of patients was 66 years (range 26 to 84), 31 male and 26 female subjects (m: f = 1,2 :1). Gastric cancer was classified according to Laurén into diffuse type (27 patients) and intestinal (30 patients). Paraffin embedded specimens were cut at 3 μm serial sections and placed on glass slides. They were deparaffinized and stained using hematoxylin and eosin. After pretreatment with EDTA immunostaining was performed with a monoclonal antibody against PDGFR-β (R&D Systems, dilution 1:20) and a polyclonal antibody against CD117 (WAK-Chemie, Berlin, Germany, dilution 1:2000) as primary antibody. Biotinylated anti-mouse IgG/ anti-rabbit IgG (Vector Laboratories, dilution 1:200) was administered as secondary antibody. Immunoreactions were visualized with the avidin biotin complex method, applying a Vectastain ABC alkaline phosphatase kit. The biotinylated secondary antibody, the avidin-horse-radish-peroxidase conjugate, and the basic DAB solution were applied, according to the manufacturer's instructions. All specimens were counterstained with hematoxylin. Primary antibodies were omitted for negative controls.

### Patient eligibility

This multicenter open label phase I study was approved by the ethics committees of the participating centers and registered with the European Clinical Trials Database (Eudra-CT 2006-005792-17) and the Clinical Trials Database (NCT 00601510). All enrolled patients gave their written informed consent. Inclusion criteria were defined: histologically confirmed advanced esophageal or gastric adenocarcinoma, presence of at least one measureable lesion according to RECIST criteria, adequate hematopoietic, hepatic and renal function – defined as: white blood cell (WBC) count ≥ 3000/μl, absolute neutrophil count (ANC) ≥ 2000/μl, platelets ≥ 100000/μl, hemoglobin level ≥ 9.0 g/dl, total bilirubin < 2 times the upper limit of normal (ULN) , SGOT and SGPT < 2.5 times the UNL (or < 5 x ULN if hepatic metastases are present), glomerular filtration rate (GFR) ≥ 60ml/min, Eastern Cooperative Oncology Group (ECOG) performance status ≤ 2. Exclusion criteria were: Any other active primary malignancy, severe uncontrolled medical illness, cardiac insufficiency (New York Heart Association III–IV), chronic liver diseases, known brain metastases, known diagnosis of human immunodeficiency virus (HIV) infection, known dihydropyrimidine dehydrogenase-deficiency, concurrent use of Sorivudin or related substances, previous radiotherapy to ≥ 25% of the bone marrow or major surgery within 2 weeks before study entry.

### Treatment design

This trial was designed to assess safety and tolerability of imatinib combined with chemotherapy in order to determine the maximum tolerable dose (MTD). Patients received imatinib and chemotherapy with cisplatin and capecitabine or cisplatin and 5-FU/Leucovorin according to the treatment regimes presented in Table 1. Therapy regimen was chosen according to the patients preferences for infusional 5-FU or orally administered capecitabine. Imatinib was administered orally as a single dose starting on day −4 and continued throughout the complete treatment cycle. The therapy regimen related to a dose escalation design with cohorts of three to six patients according to the Fibonacci scheme. Imatinib dosage started at 300 mg and the maximum dose level to reach was 800 mg. If none of three patients had dose limiting toxicity (DLT), dose was escalated in 100 mg steps cohort-to-cohort. If one of three patients experienced a DLT, three more patients were included in this cohort. Further dose escalation was only permitted if not more than one of six had DLT. The MTD was the highest dose of imatinib that resulted in DLT in fewer than one in 3 or 2 in 6 patients per cohort. Only toxicities during the first three cycles were considered for defining the MTD. Combination therapy continued until best response, evidence of progressive disease, unacceptable toxicity, death or withdrawal of patients consent. The preliminary antitumor activity was defined as a secondary objective.

**Table 1:** Chemotherapy regimens

	**Substance**	**Dose (mg/m**^**2**^**)**	**Application**	**Schedule**
**PLF** q d 50	cisplatin	50	1 hr infusion	d 1, 15, 29
	5-fluoruracil	2000	24 hr infusion	d 1, 8, 15, 22, 29,36
	leucovorin	500	2 hr infusion	d 1, 8, 15, 22, 29,36
**XP** q d 22	cisplatin	60	1 hr infusion	d 1
	capecitabine	1250	bid p.o.	d 1 -14

### Toxicity and efficacy assessment

Safety and toxicity assessment was performed by regular patient interviews, laboratory tests and physical examinations. Potential dose limiting toxicities were graded according to the National Cancer Institute Common Toxicity Criteria (NCI-CTC VERSION 3.0). Dose limiting toxicity (DLT) was defined as grade 3 neutropenia with fever or infection; grade 4 neutropenia persisting ≥ 7 days; grade 3 thrombocytopenia with bleeding or grade 4 thrombocytopenia > 7 days; any non-hematological toxicity grade 3 or 4 except alopecia, nausea and vomiting; increase in urinary retention parameters ≥ grade 2; peripheral sensory neuropathy ≥ grade 3. Relation between study drug and toxicity was evaluated with respect to the differences of the side effects of fluoruracil/leucovorin and capecitabine. Dose modifications were conducted as follows: If a patient experienced a non-hematological ≥ grade 2 toxicity administration of the study drug and chemotherapy was withheld until toxicity has resolved to ≤ grade 1. Then chemotherapy and imatinib were resumed at the same daily dose. If grade 2 toxicity recurred dose of the suspected underlying drug was reduced according to a predetermined scheme. If the criteria for continuation of the chemotherapy were not fulfilled after 2 weeks of dose modification or treatment break the patient was withdrawn from the trial. If hematological toxicity ≥ grade 3 occurred treatment had to be interrupted until toxicity had resolved to ≤ grade 1. Imatinib and chemotherapy proceeded at the same dose if toxicity resolved within two weeks. If the toxicity ≥ grade 2 persisted or recurred imatinib had to be withheld and therapy had to be adapted referring to the study scheme. No dose reductions were performed for ≥ grade 3 anemia.

Antitumor activity was evaluated as a function of objective tumor response. For efficacy assessment CT or MRI scans were performed before start of study treatment, after 6 weeks and thereafter every 12 weeks until disease progression or study withdrawal from any other cause. Responses were defined according to RECIST criteria and classified according to the WHO-criteria.

## Results

### Expression of PDGFR and c-kit in gastric cancer

**Figure 1:**
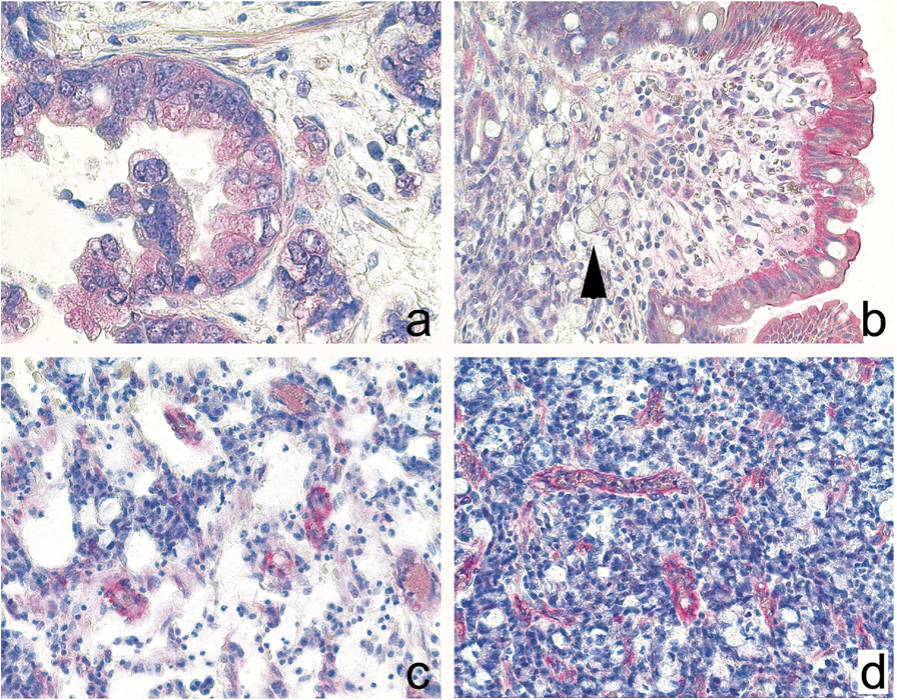
**PDGF-immunohistochemistry.** Expression of PDGFR-β in gastric cancer specimens: Intestinal type (**a**) gastric cancer expressed more commonly PDGFR-β, while diffuse type (**b**) often showed no expression. Note strong expression of PDGFR-β in the surface epithelium (**b**). Interestingly, PDGFR-β, was also found in myocytes of tumor blood vessel (**c**, **d**). Anti- PDGFR-β, hematoxylin counterstain; Original magnifications: x400 (**a**), x200 (**b, d**).

The expression and spatial distribution of PDGFR-β was investigated by immunohistochemistry of tissue specimens obtained by surgery from 20 patients. Immunostaining for PDGFR-β was found in each specimen. PDGFR-β was detected in the tumor cells of 16 patients more commonly in intestinal type [9 (100%) patients] than in diffuse type [6 (55%) patients]. A cytoplasmic staining of less than 10% of the tumor cells was found in 6 cases, a staining of 10-50% in three, and a staining of greater than 50% was found in 7 tumors. Apart from tumor cells, gastric surface epithelium expressed PDGFR-β in 10 patients, and smooth muscle cells of the muscularis mucosae or muscularis propria in 18 patients. Interestingly, PDGFR-β was expressed also from moderately to strongly by myocytes of vessel walls in 19 patients (Figure 1).

**Figure 2:**
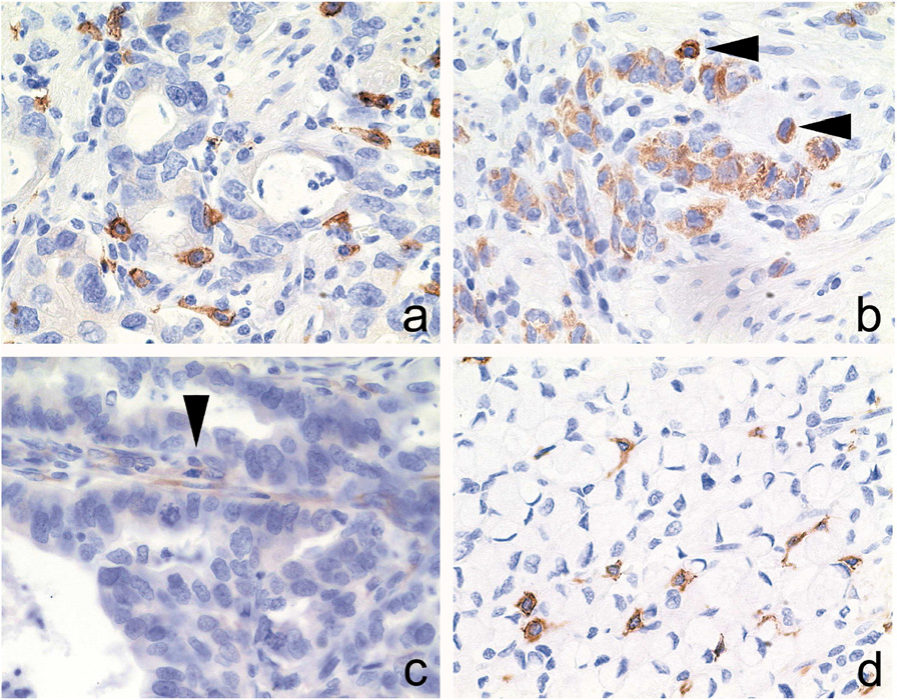
**C-kit-immunohistochemistry.** Expression of CD117 in gastric cancer specimens: Intestinal type (**a**, **b**-arrowhead) and diffuse type (**d**) gastric cancer specimens enclosed CD117-immunoreactive mast cells. Occasionally was CD117 expressed by tumor cells (**b**) and stromal cells (**c**-arrowhead). Anti-CD117, hematoxylin counterstain; Original magnifications: x400.

To determine the expression and spatial distribution of CD117 immunohistochemical analysis was carried out in neoplastic and non-neoplastic tissue specimens from 37 patients. CD117 immunoreactivity was present in each specimen, it was found in every case in mast cells and in cells of Cajal. Tumor cells stained for CD117 in 4 (11%) patients. In 3 cases less than 10% of the tumor cells expressed CD117 and in a single case 10-50% of the tumor cells. In 3 tumor specimens a weak expression of CD117 was found in few cells of the tumor stroma (Figure 2).

### Patients

From March 2008 to November 2010 39 patients were enrolled in the study. Two patients dropped out due to a rapid worsening of their general condition before starting treatment. One patient decided to have chemoembolisation of liver metastases and one patient withdrew consent for personal reasons before first dosage. Thus 35 patients were eligible for the study and assigned to 5 treatment cohorts. The median age was 61 years (range, 39–76 years), and 21 (60%) of patients had an ECOG PS of 0. For 15 (43%) patients without evidence for distant metastases therapy was started as neoadjuvant treatment. The remaining 20 (57%) patients received palliative chemotherapy intent due to distant metastases, an unresectable primary tumor or cancer manifestation as a relapse of malignancy. One patient initially considered unresectable underwent surgery after notably shrinkage of a paraesophageal lymph node metastasis. Thirty-five patients were assessable for toxicity and safety analysis, 25 (71%) patients were assessable for efficacy. Baseline characteristics are summarized in Table 2.

**Table 2:** Patient baseline characteristics (n = 35)

**Characteristics**	**N = 35**
Median age (range), years	61 (39–76)
Male/female	27 / 8
**ECOG / PS**	
ECOG 0	21
ECOG 1	13
ECOG 2	1
**Primary tumor site**	
Gastroesophageal junction	16
Stomach	19
**Histology**	
well / moderately differentiated	6
poorly differentiaited / signet- ring cell type	21
**Disease status**	
Locally advanced	19
Metastatic	22
**Metastatic sites**	
Liver	12
Lymph nodes	11
Peritoneum	4
Lung	5
Bone	2
Other	1

ECOG, Eastern Cooperative Oncology Group.

PS, Performance status.

### Safety, tolerability and MTD

The 35 eligible patients were treated in 5 cohorts at 5 dosage levels of imatinib. At imatinib dose level 1 (300mg) within 3 patients one DLT was observed consisting of immediate gastrointestinal intolerance with grade 3 nausea and anorexia. According to the 3-patient-cohort study design three more patients were included in this dosage group and no further DLT emerged. The imatinib dose was escalated via three more cohorts without any additional DLT encountered.

At dose level 5 (700mg imatinib) two gastric perforations occurred. The first patient (73 year old, male) with gastric cancer experienced this severe adverse event 4 weeks after start of therapy. Imatinib intake had lasted only 7 days until this time point because recurrent moderate toxicities (nausea, diarrhea) were observed and demanded – together with the worsening general condition- repeated hospitalization and treatment breaks. After appearance of the first symptoms gastric perforation was diagnosed immediately and emergency surgery was performed. Initially, the patient recovered as expected after surgery but then several cerebral seizures occurred. Computed tomography scan revealed extensive cerebral metastases. It was decided not to continue with anticancer therapy in view of the fatal prognosis and the patient died shortly thereafter while receiving palliative care. The second case of gastric perforation developed in a 55 year old female gastric cancer patient 7 days after start of treatment with 7 days of imatinib intake completed. Emergency surgery of this patient was performed in a local hospital and the patient recovered without sequelae.

Regarding these dose-limiting events imatinib dose level 4 (600 mg) was defined as the maximum tolerated dose (Table 3). All additionally enrolled patients were assigned to this dose level until the number of patients evaluable for response analysis was reached.

**Table 3:** Dose limiting toxic effects by dose level

**Dose level (mg)**	**n**	**Dose limiting toxicity (n)**
300	6	nausea, anorexia (1)
400	3	-
500	3	-
600	11	-
700	2	gastric perforation (2)

Median treatment duration was 14 weeks (range 1–24). Most of the adverse events were grade 1 (65%) and 2 (21%). Major grade 3 (14%) and grade 4 (1%) toxicities were nausea (6%), anemia (6%), fatigue (3%) and upper GI hemorrhage (3%). There was no difference depending on the chosen chemotherapeutic regimen (fluoruracil or capecitabine). Dosage reduction of chemotherapy was necessary in 7% of the administered cycles without notable correlation to the dose level of imatinib. No patient died as a consequence of treatment related toxicity. Toxicities are presented in Table 4.

**Table 4:** Toxicities according to the National Cancer Institute Common Toxicity Criteria

**Dose level (mg)**	**1 (300)**			**2 (400)**			**3 (500)**			**4 (600)**			**5 (700)**			**Total**		
**n**	**6**			**3**			**3**			**11**			**2**			**35**		
**Toxicity grade**	**1/2 3 4**			**1/2 3 4**			**1/2 3 4**			**1/2 3 4**			**1/2 3 4**			**1/2 (%)**	**3 (%)**	**4 (%)**
***Hematologic toxicity***																		
Anemia	-	-	-	-	1	-	-	-	-	1	1	-	-	-	-	1 (2,9)	2 (5,7)	**-**
Neutropenia	-	-	-	1	-	-	1	-	-	1	1	-	-	-	-	3 (8,6)	1(2,9)	-
Thrombocytopenia	-	-	-	-	-	-	2	-	-	1	-	-	-	-	-	3 (8,6)	-	-
***Nonhematologic toxicity***																		
Nausea	5	-	-	1	-	-	1	-	-	10	2	-	-	-	-	17 (48,6)	2 (5,7)	-
Vomiting	1	1	-	1	-	-	-	-	-	6	-	-	-	-	-	8 (22,9)	1(2,9)	-
Stomatitis	2	-	-	-	-	-	-	-	-	2	-	-	-	-	-	4 (11,4)	-	-
Diarrhea	3	-	-	-	-	-	-	-	-	14	-	-	-	-	-	17 (48,6)	-	-
Gastrointestinal perforation	-	-	-	-	-	-	-	-	-	-	-	-	-	-	2	-	-	2 (5,7)
Gastrointestinal bleeding	-	-	-	-	-	-	-	-	-	1	1	-	-	-	-	1(2,9)	1(2,9)	-
Constipation	1	1	-	-	-	-	-	-	-	7	-	-	-	-	-	8 (22,9)	1(2,9)	-
Edema / fluid retention	1	-	-	-	-	-	2	1	-	5	-	-	-	-	-	8 (22,9)	1(2,9)	-
Fatigue	-	-	-	1	-	-	-	-	-	10	1	-	-	-	-	11 (31,4)	1(2,9)	-
Fever	-	-	-	1	-	-	-	-	-	1	-	-	-	-	-	2 (5,7)	-	-
Pain	3	-	-	-	-	-	1	-	-	3	2	-	1	-	-	8 (22,9)	2 (5,7)	-
Sensory neuropathy	1	-	-	-	-	-	-	-	-	3	-	-	-	-	-	4 (11,4)	-	-

### Activity

First efficacy assessment was performed 6 weeks after start of study treatment. According to the study protocol only patients who received imatinib for at least three weeks are assessable for efficacy, leaving 25 out of 35 eligible patients for analysis. Response evaluation at 6 weeks revealed partial response (PR) in 27%, stable disease (SD) in 43% and progressive disease (PD) in 13% of the assessable patients. For the second response evaluation 12 weeks later 11 patients were assessable. The majority of those achieved stable disease (55%), partial response was affirmed in 27% and 18% had progressive disease. At both evaluations there was no difference in activity concerning cancer localisation (esophageal or gastric cancer) or chosen chemotherapeutic schedule (cisplatin/capecitabine or cisplatin/5-fluoruracil/leucovorin). For 13 (37%) patients who abandoned study treatment due to progressive disease median time to progression was 19 weeks (range 6–57). The median overall survival for these patients was 59 weeks (range 22–103).

## Discussion

Improvement of the therapeutic options for patients with advanced esophageal and gastric adenocarcinoma is an ongoing challenge considering the poor prognosis. So far, the effectiveness of chemotherapy in a preoperative as well as in a palliative situation has been demonstrated by several trials [2,24]. The superiority of multidrug regimens over mono-therapies has been proven. Therefore, great efforts are currently made to enhance the anticancer effects of chemotherapy. The integration of molecular targeted agents into common treatment schedules may be a promising approach.

In this trial, safety and tolerability of imatinib in combination with 5-FU and platin-based combination chemotherapy were evaluated to determine the MTD. Analysis of the toxicity data from treatment of 35 patients in a 3-patient-cohort dose-escalating design revealed 600 mg imatinib as the MTD. This finding is similar to the results of the study by Al-Batran et al., who had examined imatinib combined with 5FU/leucovorin chemotherapy in gastrointestinal cancers [19]. The observed side effects were moderate and most of the toxicities observed were related to chemotherapy or progression of tumor disease. Grade 3 and grade 4 toxicities are comparable to those published in previous trials with 5-FU and platin-based treatments. Unexpectedly no grade 3 and 4 diarrhea occurred and only one significant case of edema or fluid retention was registered.

Two gastric perforations constituted the most serious events of this trial. Emergency surgery in both patients provided immediate care and no life-threatening consequences were observed. However, the reasons and underlying biological mechanisms for the perforations remain unclear. A particular strong effect on the tumor tissue at the dosage level of 700 mg imatinib could be discussed. There are several cases of perforations during imatinib therapy referred to in the literature, mostly due to a rapid response of the primary tumor or the metastases [25]. But in both cases perforation emerged already after 7 days of imatinib intake and therefore after only one course of chemotherapy. One patient experienced perforation 4 weeks after therapy start with repeated interruptions of treatment as described before. Histopathological analysis of the resected specimens showed poorly differentiated tumors with transmural infiltration in both cases which is regarded to be a negative prognostic factor [26]. These findings may argue that the primary tumors were the reason for the perforations, but final determination was impossible. In addition, increased uptake of chemotherapy into the cancer cells mediated by imatinib could be a further reason for the observed large impact of the combination treatment on primary gastric cancer.

Regarding activity the combination of imatinib and chemotherapy did not completely meet the expectations. Compared with response rates of about 38% under treatment with cisplatin/5-FU/leucovorin reported on in former studies [27] the results of this trial lag slightly behind. In combination with the observed perforations in two patients at a very early point of treatment the observed reduced response rate may indicate that the combination of chemotherapy with imatinib leads to a fast and dramatic reduction of cancer cells, however, the cancer cells ultimately develop resistance mechanisms early which counteract the observed initial tumor shrinkage. Nonetheless, this phase I study was designed to determine the maximal tolerated dose. Considering the small number of patients assessable for response, no final conclusions can be made.

## Conclusions

In summary, the combination of imatinib and cisplatin-based chemotherapy was well tolerated. A dose of 600 mg imatinib daily was defined as the maximum tolerated dose in this trial. Anticancer activity of this combination was observed transiently and early. Therefore, this treatment regimen may provide benefit especially in the preoperative setting. However, further investigations – particularly of a larger group of patients - are required to confirm the amplification of chemotherapy effects by imatinib in this and other cancers.

## Competing interests

A. Krause is an employee of Novartis. All other authors declare no conflicts of interest.

## Authors’ contributions

The authors` contributions are the following: MM contributed with the implementation of the study protocol, analyses of the findings and writing of the manuscript. MPE conceived the basic study concept and the design of the protocol in addition he contributed with interpretation of the findings and editing the manuscript. KB performed the histopathological analyses. NS, SB and AK contributed with the interpretation of the final results and editing the manuscript. RC and his research team performed and optimized the immunohistochemical analyses. RH and RMS contributed with revising the manuscript and discussing appropriate judgment of the results. All the authors read and approved the final manuscript.

## Pre-publication history

The pre-publication history for this paper can be accessed here:

http://www.biomedcentral.com/1471-2407/12/587/prepub
